# A wellbeing program to promote mental health in paediatric burn patients: Study protocol

**DOI:** 10.1371/journal.pone.0294237

**Published:** 2024-02-15

**Authors:** Nicole Wickens, Lisa McGivern, Patricia de Gouveia Belinelo, Helen Milroy, Lisa Martin, Fiona Wood, Indijah Bullman, Elmie Janse van Rensburg, Alix Woolard

**Affiliations:** 1 Telethon Kids Institute, Perth Children’s Hospital, Nedlands, Western Australia, Australia; 2 The University of Western Australia, Crawley, Western Australia, Australia; 3 Burn Injury Research Unit, The University of Western Australia, Crawley, Western Australia, Australia; 4 Burn Service of Western Australia, Perth Children’s Hospital, Nedlands, Western Australia, Australia; Federal University of Paraiba, BRAZIL

## Abstract

**Background:**

One of the most traumatic injuries a child can experience is a severe burn. Despite improvements in medical treatments which have led to better physical outcomes and reduced mortality rates for paediatric burns patients, the psychological impact associated with experiencing such a traumatic injury has mostly been overlooked. This is concerning given the high incidence of psychopathology amongst paediatric burn survivors.

**Objectives:**

This project will aim to pilot test and evaluate a co-designed trauma-focused intervention to support resilience and promote positive mental health in children and adolescents who have sustained an acute burn injury. Our first objective is to collect pilot data to evaluate the efficacy of the intervention and to inform the design of future trauma-focussed interventions. Our second objective is to collect pilot data to determine the appropriateness of the developed intervention by investigating the changes in mental health indicators pre- and post-intervention. This will inform the design of future interventions.

**Methods:**

This pilot intervention study will recruit 40 children aged between 6–17 years who have sustained an acute burn injury and their respective caregivers. These participants will have attended the Stan Perron Centre of Excellence for Childhood Burns at Perth Children’s Hospital. Participants will attend a 45-minute weekly or fortnightly session for six weeks that involves building skills around information gathering, managing reactions (behaviours and thoughts), identifying, and bolstering coping skills, problem solving and preventing setbacks. The potential effects and feasibility of our intervention will be assessed through a range of age-appropriate screening measures which will assess social behaviours, personal qualities, mental health and/or resilience. Assessments will be administered at baseline, immediately post-intervention, at 6- and 12-months post-intervention.

**Conclusion:**

The results of this study will lay the foundation for an evidence-based, trauma-informed approach to clinical care for paediatric burn survivors and their families in Western Australia. This will have important implications for the design of future support offered to children with and beyond burn injuries, and other medical trauma populations.

## Introduction

### Psychological and psychosocial outcomes following a paediatric burn injury

Burn injuries are one of the leading causes for hospitalisation amongst Australian children and adolescents [1]. These injuries are profoundly traumatic for children, due to the pain of the acute injury, subsequent laser and scarring treatments, and procedures that continue across prolonged periods [2–4]. Although improvements in medical treatments have led to better physical outcomes and reduced mortality rates for paediatric burns patients, the psychological impact associated with experiencing such a traumatic injury has mostly been overlooked, particularly within the routine care context [5–9]. This is despite a wealth of studies showing that children and adolescents who experience a burn injury are at increased risk for both short- and long-term psychopathology [10].

Although some children who sustain a burn injury do not experience later mental health difficulties, others can experience a range of psychosocial problems. In a recent systematic review, Woolard et al [4] found that this population are highly vulnerable to a range of mental health problems such as anxiety, traumatic stress symptoms, depression, and emotional issues. Although there was variability with some mental health outcomes such as depression, emotional issues, self-esteem, and quality of life, the authors found that the most consistently reported mental health concerns in children who have sustained a burn injury were increased anxiety symptoms and traumatic stress [4]. It has been reported that children who have sustained a burn injury are at greater risk of having an anxiety disorder (28%) compared to uninjured children (6.9%) [4, 11, 12]. Factors that have been associated with high rates of anxiety in paediatric burns patients include hospitalisation, pain related to medical procedures, the injury itself, wound dressings, procedural fear, appearance concerns, and gender (higher anxiety observed in female children) [4]. Disruption to schooling and other lifestyle factors such as missing out on sporting activities have also shown to have a big impact on the child [13]. Furthermore, Woolard et al [4] found mixed results regarding the association between severity of the burn and later psychosocial outcomes [4]. For example, anxiety and depression were found to be associated with pain severity and intensity, whereas other psychological outcomes showed no direct association with severity [4]. However, another study found that burn size did not impact mental health outcomes and did not necessarily impact perception of pain [14]. Bakker et al (2013) examined stress symptoms in children who have sustained a burn injury and found that approximately 8% of children required treatment for an Acute Stress Disorder (which is comparable to 6% to 30% of people who have experienced other types of trauma [15–18]) [10]. Further studies with paediatric burn patients have examined chronic stress symptoms and found that 11.7% of children [19] and 13.2% of toddlers [20], met the full criteria for Post-Traumatic Stress Disorder (PTSD). Concerningly, these rates increase to anywhere between 15% to 66.7% when looking at subthreshold levels of stress (at least one cluster of trauma-based stress symptoms such as re-experiencing the event, avoidance, emotional numbing or increased arousal) [19].

In addition to the trauma experienced by the child, the impact of a paediatric burn on the family can also be traumatic [21]. A commonly reported outcome in caregivers following their child’s injury is feelings of guilt associated with the injury [21, 22]. Feelings of guilt have been positively associated with traumatic stress and anxiety in caregivers, which can be maintained for many years after the injury event [23]. Some families experience increased parenting stress and poorer family functioning due to a barrage of hospital stays or appointments, providing dressing changes and managing scar treatments [19]. Given this increased carer burden, families of paediatric burn patients can experience considerable distress that can have a negative impact on the family’s overall functioning [22]. Research has found that a high proportion of caregivers with a child who has sustained a burn injury were diagnosed with anxiety (33% to 69%) and depression (22% to 44%) [22], which is concerning given that parental anxiety is a risk factor for anxiety disorders in their offspring [24].

It is important to recognise that not all paediatric burn patients and their families experience negative mental health outcomes post-injury. To gain an understanding of why some patients adapt better to the challenges of sustaining a burn injury than others, Woolard et al [25] conducted a systematic review and meta-analyses to identify the barriers, enablers and interventions that lead to psychological recovery after sustaining a burn. This study found that having strong social support, (i.e., positive relationships with family, friends, and community) as well as spirituality were both key factors that enabled resilience and Post Traumatic Growth (PTG) in burn patients. Resilience was found to be enabled through personality factors such as optimism, and positive skills-based factors including problem-solving, social competence and autonomy [25]. Additionally, PTG was facilitated by coping mechanisms (e.g., acceptance of the injury), narrative restructuring to positively reframe the accident, and extraversion [4, 25]. This review identified the need for more research in the underlying mechanisms of both resilience and PTG, especially in child and adolescent populations who make up a large demographic for burn injuries [25].

#### Existing interventions for medical trauma

The risk of adverse mental health outcomes among paediatric burn patients makes the implementation of timely and effective interventions for these children imperative. Several trauma-informed interventions have been developed and tailored to other populations, with some treatment types involving exposure to the original traumatic event [26]. For example, Narrative Exposure Therapy (NET) [27] enables patients to retell their trauma-narrative, typically while engaging in trauma-focused cognitive-behavioural therapy (TF-CBT). The retelling of the trauma-narrative allows patients to habituate to their traumatic experience and facilitates the guided analysis of painful emotions, leading to emotional recovery from the trauma. Studies have validated the efficacy of narrative therapy in traumatised child populations, demonstrating that it is not only an effective model of treatment [28], but can also be superior to alternative interventions [29]. Another effective trauma-focused intervention is the Skills for Psychological Recovery (SPR) intervention, developed by the National Child and Traumatic Stress Network and National Centre for PTSD’s [30]. Based on extensive research surrounding the most common emotional and behavioural reactions following natural disasters, SPR is a brief and effective intervention designed to promote healthy psychological recovery after traumatic experiences [31]. Although effective, trauma-focused therapies such as TF-CBT and SPR have not been trialled in paediatric burn populations.

Despite evidence demonstrating that a burn injury can be a traumatic event that can impact mental health, mental health is often overlooked in post-burn recovery. There are a few psychosocial interventions which seek to fill this gap, but most are focused on distraction from painful procedures rather than focusing on the recovery following the burn [32]. The authors of this systematic review found that: a) existing interventions only draw from singular techniques (e.g., CBT and virtual reality [VR]) to deliver psychosocial care which has shown mixed results, and b) there is no individual technique which suits the needs of all participant demographics. The current intervention fills this gap through a co-design process with community stakeholders (caregivers, young people, researchers, and clinicians) who have determined that a combination of individual techniques (TF-CBT, SPR and NET) was important to include in this pilot intervention.

Co-design is participatory research that actively involves stakeholders in the development of an intervention to meet the needs of people it seeks to assist [33]. This collaborative approach can improve the creative process of developing an intervention, improve service delivery, and acceptability amongst its users [34]. Co-designed interventions have previously been used to map children and families’ emotional journey following a burn injury [35], as well as in the development of other mental health interventions following childhood medical trauma [36]. The current paper describes the process used for community consultation and collaborative adaptation of the proposed intervention, and the piloting of this intervention with children and young people who have experienced a burn and clinicians. This pilot study can help us understand if the methods and procedures are feasible and if the sessions designed are appropriate and accepted by participants. At a broader level, this study will provide paediatric burn health services and clinical teams, a program that adopts a trauma-informed approach to care to recognise and respond to trauma and ease the recovery journey. Early intervention is needed to reduce the impact of poor mental health and allowing for more effective healthcare pathways for paediatric burn patients.

## Objectives

The project seeks to further elucidate sources of stress and trauma that accompanies a burn injury and to develop a much-needed trauma-focused psychosocial intervention through co-design to maximise its impact in this population. We also seek to pilot test and evaluate this intervention to support resilience and promote positive mental health recovery in children and adolescents who have sustained a burn injury. The specific objectives of the intervention are to:

1. Undertake preliminary analyses to explore the association of mental health indicators with burn characteristics and demographics.
2. Collect pilot data to determine the appropriateness of the developed intervention by investigating the changes in mental health indicators pre- and post-intervention.

## Methods

### Study design

This study is a non-randomised, pre-post, cohort study which seeks to determine the potential effects of a psychosocial intervention. We use convenience sampling, with data collected from age-appropriate questionnaires at four time points: Time 1 (T^1^): baseline assessments; Time 2 (T^2^): post intervention assessments; Time 3 (T^3^): 6-month follow up assessments; Time 4 (T^4^): 12-month follow up assessments. Randomisation and blinding are not relevant in this pilot study due to the single intervention arm, where intervention’s absolute effect on the participants across time is the primary interest to determine the appropriateness of the intervention content [37].

### Ethics

The study has received ethics approval from the WA Child and Adolescent Health Service (CAHS) Human Research Ethics Committee (HREC) and Research Governance Office (RGS4669). Any intervention protocol modifications will be approved by these committees and communicated to the participants.

### Stage 1: Co-design process

Caregivers and adolescents were invited to participate in an interview or focus group and were provided the opportunity to (1) discuss the traumatic impact of a paediatric burn injury and (2) evaluate the proposed intervention. Participants were recruited from the Stan Perron Centre of Excellence for Burns and the Perth Children’s Hospital (PCH) burns outpatient clinic. The following groups took part in this co-design process: caregivers (n = 11, dyadic interview = 1, interviews = 9) of children aged 4–12 years who have sustained a burn injury and adolescents (n = 7, interviews = 7) (aged 12 years and older) who have sustained a burn injury. In addition, clinicians (n = 5, focus group = 1) from the Stan Perron Centre of Excellence for Childhood Burns and/or the PCH burns outpatient unit participated in a focus group to provide feedback on the design and structure of the intervention. Due to COVID-19 restrictions at PCH, all interviews and focus group were conducted online as semi-structured interviews using the Microsoft Teams platform.

This co-design process was conducted: (1) to provide valuable insight into the traumatic nature of acute paediatric burns from the perspective or caregivers and adolescents and (2) determine the acceptability of our intervention from the perspective of adolescents, caregivers and clinicians. Audio was recorded during focus groups and interviews to ensure all participant contributions were documented. To explore the traumatic nature of a paediatric burn on the family from a child’s [13] and caregiver’s perspective [21], recordings were transcribed and de-identified. Furthermore, to determine the acceptability of the proposed intervention, the intervention outline and resource materials were presented to all participants, after which, they were asked a series of questions to provide feedback for the intervention. All intervention amendments and adaptations suggested by caregivers, adolescents and clinicians were agreed upon by the end of the collaborative design session. Participants were given the opportunity to view the final developed intervention upon request.

### Stage 2: Intervention

#### Participants

*Participant eligibility criteria*. Eligibility will include school aged children aged 6–17 years who have sustained a burn injury and attended the Stan Perron Centre of Excellence for Childhood Burns at PCH in the previous 12 months, and their caregivers. Participants must be fluent in speaking and reading the English language (due to limited funding for interpreters and translators, alongside a lack of validated measures for other languages). Participants must also have access to an internet connected device (e.g., computer, tablet, mobile phone) to facilitate the intervention sessions and study surveys.

*Exclusion criteria*. Children aged 0–5 years will be excluded from the study as sessions for children aged 0–5 would be a family intervention involving the caregiver, rather than a child-directed intervention. Children presenting with an intentional burn injury (i.e., burn injuries that are self-inflicted or inflicted by others to cause harm [38]), or who have a diagnosed cognitive impairment (e.g., traumatic brain injury) that means they are unable to attend/understand the intervention content will be excluded from this study. Caregivers who have a known substance abuse issue or are unable to appropriately engage in the intervention due to pre-existing severe psychological distress will also be excluded. The exclusion criteria will be identified during the screening process, through discussions with the clinical and PCH burns research team and recorded patient medical notes on the Burns Information Management System (BIMS). If not previously disclosed, this information can also be identified in the demographic questionnaire provided to participants during the baseline screening process. Levels of understanding of the requirements for research participation will be determined by the completion of the consent process.

*Recruitment and consent*. Participants will be recruited by a member of the research team at their outpatient burns clinic appointment at PCH. Convenience sampling will be used to recruit participants into this pilot study from PCH due to its paediatric burn’s outpatient clinic’s proximity and accessibility for the research team. PCH oversees the management of most paediatric burn cases across WA, especially for more severe cases.

Eligible participants who are approached will be given a Participant Information Sheet (PIF), will have the project explained verbally by the researcher, and will be given the opportunity to ask any questions. Full informed consent will ensure that the caregiver understands the adequate dosage of intervention that needs to be provided to see sufficient therapeutic gains and that the caregiver provides commitment (and that of the child’s) to the process of intervention in order to minimise attrition. Informed consent will be sought from young people aged 12 years and older and their caregivers via REDCap [39]. Children aged 6–11 years old will be provided with an age-appropriate verbal explanation of the study by the researcher. The researcher who conducts the consenting process will also sign a consent form, confirming they have checked participants’ identity and have given a verbal explanation of the study to the participant. In addition to the information obtained during T^1^ (baseline assessments), caregivers will be asked to consent to their child’s medical records (obtained from the Child Adolescent Health Service [CAHS] database) being used in the current study. Specifically, the PIF asks caregivers if the researchers can access the following information about their child: location of the burn, severity of the burn, date of burn event, time spent in hospital following burn. This information will be used to further describe the demographics of study participants.

*Sample size*. There will be an active recruitment process until the proposed sample size of 40 children has been met. This is a novel intervention for paediatric burn patients, and thus similar outcome analyses have not been published, making sample size estimation difficult. Sample size calculations are not required for all pilot studies, however they must be representative of the target study population and inclusion criteria [40]. Thus, this pilot study has a sample size of 40 children, that is representative of the paediatric burns population at our study site and participant eligibility criteria. Having a sample size of 40 children will ensure this study is feasible and accounts for a 20% attrition rate.

Furthermore, one caregiver from each participant must be involved in the study. Caregivers in this study will not participate in the intervention but will complete specific questionnaires that will be self-assessed or completed on behalf of their child, for cross-informant reporting for their children’s mental health status and outcomes throughout the study.

## Overview of intervention design

The piloting of the intervention will be delivered by a trained member of the research team with experience working in mental health settings. Sessions will be held weekly or fortnightly (based on participant availability) for a total of six sessions, with each session taking approximately 45-minutes to complete. Based on findings from the co-design process and current evidence-based practice, the intervention sessions were designed to contain skills included in TF-CBT, SPR and NET. The structure and content of sessions were informed by the common principles co-existing across evidence-based trauma-focused interventions [27] including: psychoeducation (first step in treatment) [27, 41–43]; coping skills such as relaxation skills (e.g., mindfulness) [44]; creating a trauma narrative [27] (the majority of trauma-informed therapies encourage the completion of a trauma narrative) [44, 45]; cognitive restructuring [27, 46]; and creating a posttreatment plan [27]. Due to the resilience based focused, this mental health recovery program was named ‘The Wellbeing Program’.

Based on the co-design process and due to the ongoing restrictions and service changes at PCH due to the COVID-19 pandemic (such as in-person appointments moving to Telehealth), the sessions will be delivered via the video communications platform ‘Microsoft Teams’ (Version 16.2.8). The online format allows the intervention to be more accessible to patients who live a significant distance away from PCH (i.e., regional/remote). If restrictions are eased, the research team will offer face-to-face sessions with participants, which will be scheduled to roughly coincide with the child’s outpatient appointments with the burn unit at PCH. However, opting only for a face-to-face intervention will restrict the number of participants who may be willing to participate.

Participants that choose to have face-to-face sessions rather than online, will be reimbursed for parking costs. Session structure and design will remain the same. For most caregivers who participated in the co-design process, online program delivery was the preferred method due to convenience and logistics. Additionally, the total number of sessions was reduced from nine to six, and further activities were incorporated to ensure children and families remained engaged for the duration of the session. With caregivers being a crucial part of a child’s burn recovery journey, their involvement in the sessions was favoured, namely, to support a shifting in feelings of guilt and blame by assisting in their child’s support process. Furthermore, the feedback from clinicians identified the impact that paediatric burns have on the child and their caregiver, and the support required throughout their burn journey. Clinicians discussed the repetitive trauma that families undergo during dressing changes, including the physical pain the child endures and the anxiety children and caregivers go through during the hospital admission period. Thus, a key focus of this intervention is to acknowledge the challenges families face, identify how far they have come, and support them through the recovery process. Additionally, clinicians recognised how the capacity to recover is dependent on child premorbid functioning and identified the importance of screening and intervening early to provide a comprehensive overview of both physical and mental health factors. With resilience building being a core focus of the intervention, clinicians reinforced how the intervention must be tailored to suit the needs and personalities of each child. Clinicians also expressed their concerns in the suitability of session content for younger children and the limited ‘fun’ aspects, therefore content was reviewed and developed for two separate cohorts (6–12 years and 13–17 years old) and additional activities were added (e.g., games such as snakes and ladders). An intervention session plan is outlined in Table 1.

**Table 1:** Revised intervention session plans.

Session	Overview
**1. Introduction, Assessment, Information Gathering**	Session aim: To establish rapport, formulate appropriate matching of skills to presenting problems and orient the subsequent visit on the highest priority areas. Review screening measure results. Introduce the caregiver booklet: A caregiver resource is provided to include information about the child moving through various types of responses to different situations and stages of healing. This resource will enable caregivers to be aware of these processes and how to best support their child. Introduce the child booklet: The child resource will be used across the sessions to complete activities, and the caregiver resource will compliment this by providing some context on the activity and the benefit it will have on their child. The facilitator explains the program, including the “why” and “how’s”. Participant intake progress notes for ages 6–12 (see S1 Table) and 13–17 years will be used to identify the most pressing needs. The first strategy that is introduced (i.e., emotional wellbeing, interpersonal connections) will align with this. The interview structure is the same for both age groups, however the language has been modified to be age appropriate. NB: For all ages, sessions 2–5 will have age-dependent activities, however the session structure will be consistent.
**2. Managing reactions (Behaviour)**	Introduction and orientation to the session; age-dependent language and use of child booklet for discussion. Psychoeducation and link to activity depending on each age. CBT introduction and link to relevant activity. Breathing activity and reaction management strategy. Homework allocation if specified in the booklet. Summary and discussion of any points that are unclear to the child/caregiver.
**3. Managing reactions (Thoughts)**	Introduction, wellbeing check, and homework revision if required. Orientation to session. Thought introduction and link to relevant activity. Homework allocation if specified in the booklet. Summary and discussion of any unclear points.
**4. Identifying and Bolstering coping skills**	Introduction, wellbeing check, and homework revision if required. Orientation to session. Coping, avoidance and withdrawal explanation and discussion. Respective booklet used for discussion. Coping activity to support the child’s understanding of healthy and unhealthy coping processes. Homework allocation if specified in the booklet. Summary and discussion of any unclear points.
**5. Problem solving**	Introduction, wellbeing check, and homework revision if required. Orientation to session. Introduction to worrying versus problem solving. Problem solving activity. Homework allocation if specified in the booklet. Summary and discussion of any unclear points.
**6. Preventing setbacks**	Introduction, wellbeing check, and homework revision if required. Orientation to session. Discussion of previous sessions. Development a story of what the child/adolescent did/enjoyed prior to their burn. Development a story of what happened during the burn (who, what, when, where), highlighting successes (what helped). Development a story of what the adolescent wants for their future, highlighting successes (what helped after the burn; including techniques taught over the program and strengths identified to date). Reading of the story aloud. Summary and discussion of any unclear points. Questionnaires will be re-administered.

### Instruments

During baseline assessment (T^1^), a demographic questionnaire will be administered to adolescents (12–17 years old) and all caregivers to capture their age, gender, and any mental health conditions they or their child have been diagnosed with prior to and after the child’s burn injury. For children aged 11 years and younger, caregivers will capture this information on their behalf. The potential effects and feasibility of our intervention will be assessed via the selection of measures from the screening procedure with the aim of determining whether the current content of the intervention can cause change in mental health outcomes for participants [47]. Children aged 8–17 years (self-report) and caregivers (caregiver report and self-report) will complete a range of screening tools at pre- and post-intervention (T^1^ and T^2^ respectively), as well as at 6- and 12-months post-intervention (T^3^ and T^4^ respectively), which will be used as outcome measures of the intervention and to assess mental health (Table 2).

**Table 2:** Child and caregiver screening measures used in The Wellbeing Program.

Construct	Measure	Participants age range	Reliability	Items (number and response procedure)	Additional information
**Internalising and externalising behaviours**	The Strengths and Difficulties Questionnaire (SDQ) [53].	Caregiver report: On behalf of children aged 6–17 years Self-report: Children aged 11–17 years only	Test-retest reliability rho = .62 and internal consistency α = .73	25 items	The SDQ is a screening tool that can be used as a treatment outcome measure for children and adolescents [53]. The SDQ asks about attributes such as emotional symptoms (5 items), conduct problems (5 items), hyperactivity or inattention (5 items), peer relationship problems (5 items) and prosocial behaviour (5 items) [53].
**Child behavioural and emotional issues**	The Child Behaviour Checklist (CBCL) [54]	Caregiver report: On behalf of children aged 6–17 years	Test-retest = .73–0.94, internal consistency C = .63 -.97), and inter-rater reliabilities = .57 -.88	113 items Likert scale 0 (not true, as far as you know) to 2 (very true or often true)	The CBCL is used to detect changes after participating in short and long behavioural and mental health interventions [55–57]. The CBCL measures child and adolescent mental health in the areas of anxiety, depression, somatic complaints, social problems, thought problems, attention problems, rule breaking and aggressive behaviour.
**Adolescent behavioural and emotional issues**	The Youth Self-Report (YSR) [58]	Self-report: Children aged 11–17 years only	Test-retest = .67 -.91, and internal consistency (α = .55 -.95	118 items Likert scale 0 (not true) to 2 (very true or often true)	The YSR is used as a mental health outcome measure and has reported tests of significance [59]. The YSR assess the same domains as the CBCL.
**Anxiety**	The State-Trait Anxiety Inventory (STAI) [60]	Self-report: Children aged 12–17 years only Caregivers	Test-retest reliability r = .65 -.75 and internal consistency α = .86 -.95	40 items Likert scale 1 (not at all) and 4 (very much so)	STAI has 20 items that indicate how an individual feels in the present moment (state) and how they generally feel (trait). Since 1970 the STAI has been used in a range of research and clinical practices to evaluate processes and outcomes in behavioural and cognitive treatments and interventions [61].
**Anxiety**	The State-Trait Anxiety Inventory–Child report (STAI-CH) [61]	Self-report: Children aged 9–12 years only	Test-retest reliability r = .16 -.61 and internal consistency α = .78–87	40 items Likert scale 1 (hardly-ever) and 3 (often)	STAI-CH is the child version of the STAI and assesses the same domains.
**PTSD symptoms**	The Child PTSD Symptom Scale for DSM-5 (CPSS-5) [62]	Self-report: Children aged 8–17 years only	Test-retest reliability r = .80 and internal consistency α = .92	27 items Likert scale (20 items) frequency and severity from 0 (not at all) to 4 (6 or more times a week /severe). 7 functioning items (yes/no)	The CPSS-5-SR measures PTSD severity. The CPSS-5-SR has a cut-off score of 31 to determine a probable PTSD diagnosis [62].
**PTSD symptoms**	The PTSD Checklist for DSM-5 (PCL-5) [63]	Self-report: Caregivers	Test-retest reliability (r = .74 -.85) and internal consistency α = .94	20 items Likert scale 0 (not at all) to 4 (extremely)	The PCL-5 is used to monitor symptom change during and after treatment, screening individuals for PTSD and making a provisional PTSD diagnosis. Weathers et al [63] suggests using the DSM-IV criteria to determine reliable (5–10 point) and significant changes (10–20 point) [64].
**Stress**	The Perceived Stress Scale (PSS-10)	Self-report: Children aged 11–17 years old only Caregivers	Internal consistency α = .78	10 items Likert scale 0 (never) to very often (4)	The PSS-10 measures individual stress levels. It has been used as an outcome measure to identify reductions in stress scores from a 10-week stress reduction program [65] and clinical studies [66].
**Stress**	The Parental Stress Scale (PSS) [67]	Self-report: Caregivers	Test-retest reliability over a 6-week period r = .81 and internal consistency α = .83	101 items Likert scale 1 (strongly disagree) and 5 (strongly agree).	The PSS has been used to measure change in parental stress levels following individual and family interventions [68]. Thus, comparisons of mean before and after scores can be made.
**Resilience**	The Connor-Davidson Resilience Scale (CD-RISC) [69]	Self-report: Children aged 10–18 years only Caregivers	Internal consistency α = .84 -.86	10 items Likert scale 0 (not true at all) to 4 (true nearly all the time)	Many studies have used the CD-RISC to assess change in resiliency during treatment including psychotherapy [70, 71] and other interventions, such as stress-management [72–74] or resilience-building [75].

Due to age range restrictions of the selected questionnaires, children aged 6–7 years old will not need to complete any self-reported questionnaires therefore, only caregiver reports will be collected for this age range. The selected questionnaires show mixed reliability among the younger age groups and often relied on parental or researcher administration to show success which is not always logistically possible [48–50]. To ameliorate this limitation, and to complement the self-reported measure, all caregivers of children in this study will be required to complete The Strengths and Difficulties Questionnaire (SDQ) and Child Behaviour Checklist (CBCL) that asks questions and statements about their child (caregiver report). Our previous research has highlighted how paediatric burns can be inherently traumatic for caregivers, particularly mothers, and thus can adversely affect their mental health outcomes [21]. Therefore, parental screening measures are included in this study despite children and adolescents being the primary target of this intervention.

All outcome measures, excluding the SDQ, will be completed at all four timepoints (T^1^, T^2^, T^3^, T^4^) and will take approximately 30–60 minutes to complete at each timepoint. The SDQ will be completed only at T^1^, T^3^ and T^4^ timepoints as the online version of the SDQ asks responders to recall information from the “previous six months”. The SDQ will be completed on the SDQ online survey platform, and all remaining measures will be completed online via REDCap [51, 52]. Permission to use the measures has been granted by the authors and/or owners. All screening measures will be administered by a research team member under the supervision of a Child and Adolescent Psychiatrist and have received training in all the data collection and analysis procedures.

A range of valid and reliable child and caregiver directed screening measures will be collected (Table 2). Questionnaire suitability was determined based on validity, reliabilities, and responsiveness to change. Age-appropriate screening measures will be used to identify changes in a child’s internalising and externalising behaviours (SDQ), general behavioural and emotional issues (CBCL and Youth Self Report), anxiety levels (The State-Trait Anxiety Inventory [STAI] and The State-Trait Anxiety Inventory—Child report [STAI-CH)), PTSD symptoms (The Child PTSD Symptom Scale for DSM-5 [CPSS-5] and The PTSD Checklist for DSM-5 [PCL-5]), stress (The Perceived Stress Scale [PSS-10] and The Parental Stress Scale [PSS]) and resilience (The Conner-Davidson Resilience Scale [CD-RISC]) after participating in this intervention.

In addition to the assessments, participant intake progress notes will be used during the first session by the facilitator to understand the child’s most pressing needs and build rapport with the child (S1 Table). The notes will provide a thorough picture of the client needs at the time of intake including their burn experience, personal and social history, mental health history, current symptoms and concerns, previous treatment received and family environment. The intake notes will remain the same for both age groups however, the facilitator can modify the wording of the questions to suit the 13–17 year old age group (e.g., Question 1: Can you talk to me about your burn experience?).

**Fig 1:**
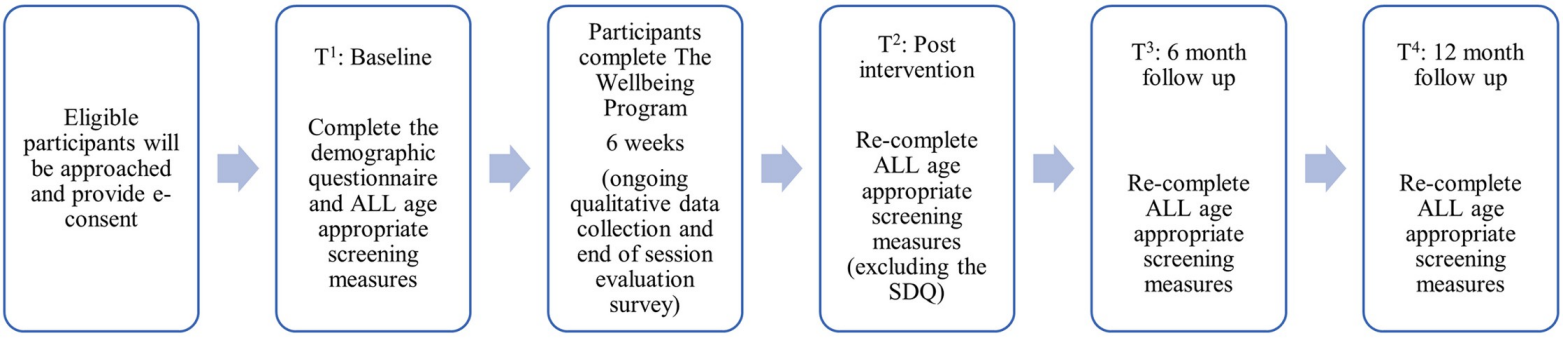
Participant involvement in The Wellbeing Program.

At the end of each session, adolescents (13–17 years) will also be sent an evaluation survey by text message to identify 1) if they enjoyed the session (Yes or No) and 2) how helpful they found the session (scale 1–5). Immediately after the final session another evaluation survey will be sent to adolescents to identify 1) what session was the most useful (ranking from 1–6) and 2) what their overall rating of the program is. This will provide children with the opportunity to provide feedback, identify participant responsiveness and ensure high quality program delivery for future interventions. Fig 1 reflects participant involvement in this intervention.

## Data management plan

This project is being conducted in compliance with the HREC study protocol, Good Clinical Practice guidelines, and the HREC application regulatory requirements. All research team members recruiting and/or delivering the sessions, have completed Good Clinical Practice training and follow a Standard Operating Procedures (SOP) document that was developed by the project investigators to provide steps and processes to undertake the project. In addition, researchers have appointed an Independent Auditor who is a Registered Psychologist to monitor the project, conduct regulatory inspections, have direct access to source data/documents to ensure quality control of data and review project safety procedures.

To ensure quality and accuracy of data, all data will be collected, de-identified and stored by the research team. All study data will be collected and managed using REDCap electronic data capture tools hosted at the Telethon Kids Institute (TKI) [51, 52]. REDCap is a secure, web-based software platform designed to support data capture for research studies. All questionnaires except for the SDQ have received permission by the authors and/or owners to replicate them onto REDCap as a survey. For simplicity, each age group will have a separate survey with all age-appropriate questionnaires and will be sent to participants via email. Once participants have completed the questionnaire, they will be scored by a member of the research team. If participant scores are of concern or reach a clinical threshold, the participant will be referred for appropriate clinical psychological or medical follow up with a General Practice practitioner. Although we have received permission to replicate the CBCL and YSR in REDCap as a survey, all participant responses will be manually entered and verified in the ASEBA Web platform to generate participant scores and reports. To ensure compliance with SDQ’s copyright policy, a separate link will be sent to participants from the SDQ online platform in which scores and reports will be generated. All SDQ scores will be manually entered on REDCap by a member of the research team. All other questionnaires will be manually scored on REDCap.

To protect the participants privacy and to maintain confidentiality, data will be anonymised by allocating a participant with a unique person identification code. Raw data collected during the intervention will be securely kept on password protected computers at TKI in a project specific database that only research team members have access to. All entries will be checked by two research assistants. Upon completion, only completely anonymised measurement data and group statistics (i.e., Confidence Intervals, mean, median, range etc.) will be made available to other researchers and reported in publications. Lastly, the TKI research team and the PCH burns research team meet once a week to ensure transparency of project planning and maintain standardised procedures across both centres.

## Data preparation and statistical analysis

All screening measures will be thoroughly checked by two research members to ensure all applicable questions have been answered. Missing data will be imputed where necessary, with the method depending on attrition rates and the degree of missing data. Scoring of all screening measures will be completed in REDCap or on the measure’s online software through syntax, which will minimise the risk of data entry errors. The quantitative data will be analysed using Stata data analysis software to determine relationships between our mental health measures and significant independent variables such as demographics (e.g., birth sex, age, age at injury) and injury characteristics (e.g., nature of the burn, size, and location) using basic descriptive analyses (Objective 1). This software will also be used to determine if the measures provide meaningful information on whether our intervention improves the mental health of children and young people who have survived a burn injury. Improvements in child and caregiver mental health (via SDQ, CBCL, YSR, STAI, STAI-CH, CPSS-5, PCL-5, PSS-10, PSS and CD-RISC) pre- (T^1^) and post-intervention (T^2^, T^3^, T^4^) will be investigated (Objective 2). Normality of all data will first be tested using Shapiro-Wilk’s test, which is ideal for sample sizes of less than 50 [76]. We will also utilize graphical presentation of the data (e.g., boxplots and histograms) to aid in determination of normality in the data [76]. If the data is normally distributed, then dependent t-tests will be employed to determine change in measure scores (e.g., changes in anxiety scores). If the data is not normally distributed, then we will employ the Wilcoxon signed-rank test instead.

Individual-based Change Statistics (IBC) such as the Standardized Individual Difference or the Reliable Change Index will also be used, as these have been shown to be effective in detecting change in clinical populations and do not require arbitrary cut-off points [77]. Additionally, we will analyse the feasibility and acceptability of the intervention via adherence and compliance to the program, attrition rates and uptake of recruitment and post-session ratings (children aged 13–17 years only). Pilot feasibility studies are able to provide information on consent rates, treatment compliance and methods of outcome measurement to assess the potential efficacy of a novel intervention [78]. The results of the analysis will enable the research team to identify participant behaviour and attitude changes, improvements in mental health, and determine if our intervention design is suitable for a future roll-out [47]. Data cleaning and statistical analyses will be performed in Stata/SPSS [79].

We intend to use restriction of enrolment and statistical analyses methods to control for confounding factors. Due to the preventative and non-clinical nature of this intervention, it was imperative that we incorporated screening measures and methods which would exclude young people and/or their carers who have existing mental health concerns. Furthermore, we intend to apply correction methods (e.g., stratification by age group) as needed to further delineate the effects of the intervention based on other participant characteristics.

## Dissemination

The findings from this study will be presented at relevant research conferences, local and national research symposiums, and seminars in collaboration with our research partners (TKI, CAHS, Fiona Wood Foundation and University of Western Australia) and other medical health services. Furthermore, dissemination of the overall findings will be communicated via a published peer reviewed academic journal. Additionally, a lay summary of the outcome of the intervention after the 12-month follow up (T^4^) will be distributed to all study participants and relevant interest groups via social media platforms.

### Status and timeline of the study

Recruitment and data collection of stage 1 occurred between February and May 2022. An HREC amendment for stage 2 was approved in September 2022 and recruitment began in November 2022. Recruitment is expected to continue until November 2023 therefore, the intervention sessions will conclude in December of 2023. Given the follow-up timepoints (T^3^ and T^4^), data collection will continue until December of 2024. Data analysis will start after all T^2^ data has been collected.

## Discussion

### Summary

The purpose of this study is to evaluate the potential effects of a resilience based mental health recovery program (The Wellbeing Program) for children and adolescents who have sustained an acute burn injury. Study results will assist in informing children, their families, and clinicians about the importance and benefits of an individualised treatment to improve psychosocial recovery following a burn injury. Several aspects of the pilot data will inform the design of the larger roll-out of this intervention within the PCH burns unit and the wider community in Western Australia. The measures will be evaluated by the research team to determine if they provide meaningful information on whether the intervention improves the mental health and resilience of children and young people who have sustained a burn injury. Furthermore, the virtual format of the sessions will provide valuable insights into the acceptability of the medium, participant engagement, and feasibility of this medium as an alternative method for offering psychological, which may improve service provision for rural and remote patients.

Stage 1 of this study resulted in a co-designed intervention that incorporated feedback from community stakeholders (adolescents, caregivers) and clinicians. The recommendations that fit within the study scope were integrated into the project and further suggestions will be considered for future intervention development.

### Strengths and limitations

The aim of this intervention is to support resilience and promote positive mental health in children and adolescents who have sustained an acute burn injury and significantly benefit routine care. Participation in this study has the potential to equip children with skills and strategies to cope during distressing situations relating to their burn injury and in the future. Since this is not a clinical intervention, rather a support program to assist families in recovery, this program can be delivered by someone without a clinical background. However, the person administering the screening tools must be suitability qualified or administer tools under supervision of a clinical psychologist or psychiatrist. Furthermore, the generalisability and accessibility of this intervention allows this intervention to be adapted for other medical trauma populations and can be accessed by children living in rural and remote areas.

Due to the nature of this pilot study, a hypothesis is not tested and therefore safety and efficacy are not evaluated [80]. Participation in this pilot research will provide valuable information for the evaluation of the intervention and will provide modifications in the planning and design of a larger efficacy trial to determine the magnitude of the effect that the intervention has on child and caregiver mental health [47]. This would also eliminate the potential influence of confounders in current protocol by means of randomization and blinding. The present protocol is limited by timeframe and funding and thus convenience sampling was deemed to be most feasible. This limits the generalisability of the study’s findings to other populations outside of Western Australia. However, progression into a randomised clinical trial may alleviate this concern.

Furthermore, this study aimed to develop a child directed intervention for children aged 6–17 years old, however 31% of all paediatric burn cases reported in the latest Burns Registry of Australia and New Zealand were aged between one to two years [81]. Thus, a family targeted intervention involving the caregivers should be the subject of further investigation. At the start of the intervention sessions all children will be provided with a child resource for ages 6–12 years old 13–17 years old. Despite sessions being tailored to meet the needs of the individual child, we do not test for intelligence or intellectual capabilities. Therefore, the facilitator needs to be reflexive and adaptive in their language to the individual capacity of the child, while only covering topics which are pre-determined within each booklet. Another limitation of this study regards the use of self-report questionnaires to assess child and caregiver mental health and resilience. Although self-reports have many advantages, they may also lead to social desirability [82]. However, the use of parental questionnaires as cross-informants to youth self-reports could ameliorate this. It would also be helpful for future studies that have the resources to look at additional behavioural or physiological measures.

Lastly, collection of physiological data from both patients and their caregivers to assess cortisol levels, for indication of stress, was not within the scope of this pilot study due to practical considerations. However, increases in cortisol levels have been associated with acute [83] and severe burn injuries [84], therefore future applications of this protocol should attempt to collect physiological data in addition to the psychological data collected at various timepoints.

### Safety considerations

Although this intervention aims to promote a positive recovery model, the risk is the exploration of emotions in a population that has experienced a recent traumatic injury. To mitigate risk of working with individuals with significant psychological distress, these individuals will be screened and referred for appropriate clinical psychological or medical follow up with a General Practice practitioner. Additionally, if it was revealed through the screening procedure, data collection, or other interpersonal communication with participants that professional psychological help or social support is required, the same risk management plan will be followed. Any concerns that the research team may have will be reported immediately to the Director of the WA Burn Service for appropriate action. Any adverse effects or unintended consequences will be immediately reported to the HREC ethics committee.

## Conclusions

The evaluation of this pilot intervention will lay the foundation for an evidence-based, trauma-informed approach to clinical care for paediatric burn patients and their families in Western Australia. The larger roll out of the intervention will be designed so that health professionals can independently run the program within their service as a routine psychological care treatment plan. Indeed, the development of an effective model of care and an evidence-based intervention for children following medical trauma can be used as a guide for other research studies.

## Supporting information

S1 Table Intake notes. (PDF)
