# Supplementary material for: A wellbeing program to promote mental health in paediatric burn patients: Study protocol
Source: PLoS One. 2024 Feb 15;19(2):e0294237. doi: 10.1371/journal.pone.0294237 (PMC10868872; doi:10.1371/journal.pone.0294237)
Supplement: S1 Table — (PDF) [file pone.0294237.s001.pdf]

Supporting information

S1 Table. Intake notes

Topic	Prompts
**Burn experience**	
**1. How did your injury happen?**	a) How often do you think about what happened?<br>b) What feelings come up for you when you think about what happened?
**Burn Related Change (Domains: Emotions, Behaviours, Physical, Academic, Social/Relational)**	
**2. Has anything changed since your injury?**<br>**(NB: Identifying and bolstering coping session)**	a) Are there any things that you can't do now?<br>ai) Are there other things you do instead?
**3. Has there been any change in the way you feel (emotion)?**	a) Can you tell me more about that?
**4. Has there been any change in the way you see yourself (psychological/self-appearance)?**<br>**(NB: Regulating appearance concerns session)**	
**5. Has anything changed with school?**	
**6. How did you deal with these changes?**<br>**(NB: Problem solving session)**	a) What helped?<br>b) What didn't help?<br>c) Did anything make you feel worse?
**Socialisation**	
**7. How would you describe school?**	a) Is this the same or different to before your injury?
**8.What is the best/worst part about school?**	a) How do you find the teachers at school?<br>b) How do you find the kids at school?<br>c) How do you find meeting new people/making friends at school?<br>ci) Is this the same or different to before your injury?
**9.What are some of the things you like to do with your free time?**	
**Mental Health**	
**10.What have been your main feelings lately?**	a) What do you do when you have these feelings? (NB: Managing reactions/thought balancing sessions)<br>ai) Have you had these feelings more than usual lately?<br>b) Who do you turn to for support when you're not feeling great?<br>bi) Why do you go to [person's name]?

**11. Have you seen any mental health professionals before [Y/N]?**	a) Do you have any conditions?<br>b) Are you taking any medication?
**12. Have you thought about hurting yourself before?**	If answer is yes: Risk contingency takes precedent
**13. Have you thought about hurting others before?**	If answer is yes: Risk contingency takes precedent
**Home environment**	
**14.Who is in your family?**	a) Who do you feel closest with?<br>b) Is there anyone you have trouble with?
**15. Do you have chores/jobs to do at home?**	
**Strength/Skills**	
**16.What are your hobbies?**	a) What do you like doing (sports/hobbies/talents) for fun?
**17.What are you good at?**	
**18.What are you looking forward to?**	a) What would you like to do in the future?
**Summary**	
**19. Is there anything that we haven’t covered that you’d like to talk about?**	
